# Supplementary material for: Designable ultra-smooth ultra-thin solid-electrolyte interphases of three alkali metal anodes
Source: Nat Commun. 2018 Apr 9;9:1339. doi: 10.1038/s41467-018-03466-8 (PMC5890267; doi:10.1038/s41467-018-03466-8)
Supplement: Supplementary file 1 — Supplementary Information [file 41467_2018_3466_MOESM1_ESM.pdf]

Description of Supplementary File

File name: Supplementary Information

Title: Designable ultra-smooth ultra-thin solid-electrolyte interphases of three alkali metal anodes

Authors: Gu et al.

Description: Supplementary Figures, Supplementary Tables, Supplementary Notes, Supplementary Methods and Supplementary References.

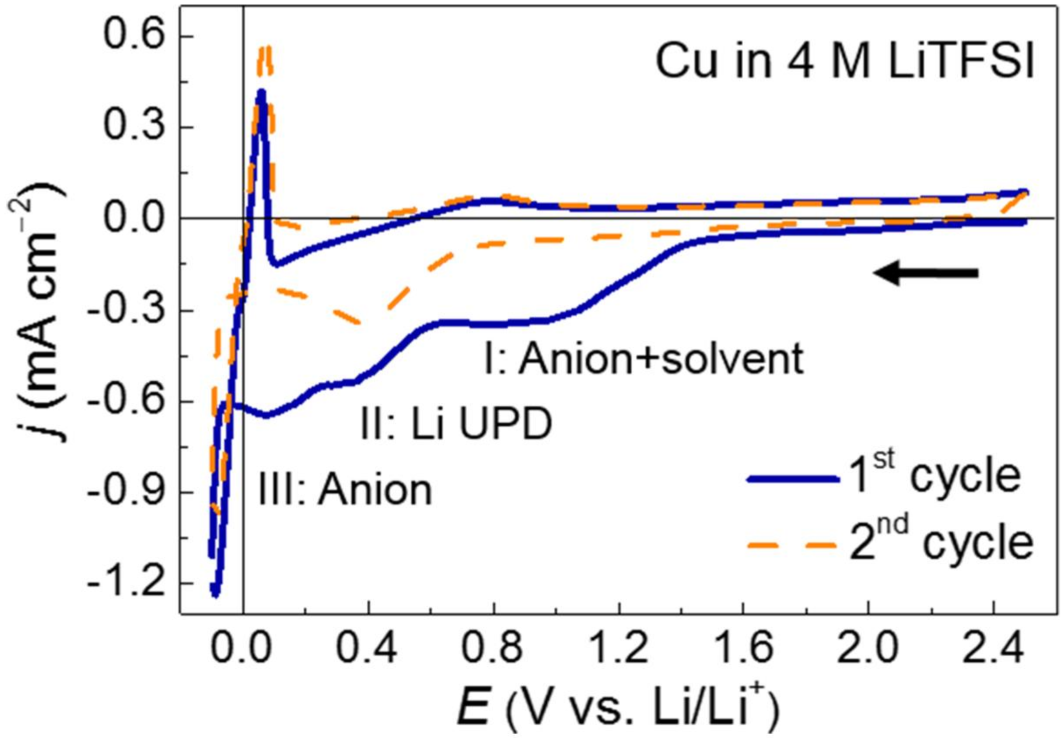

Supplementary Figure 1 | Cyclic voltammograms of Cu in 4 M LiTFSI/DME-DOL (1/1, V/V).

Scan rate: 20 mV s^{-1}.

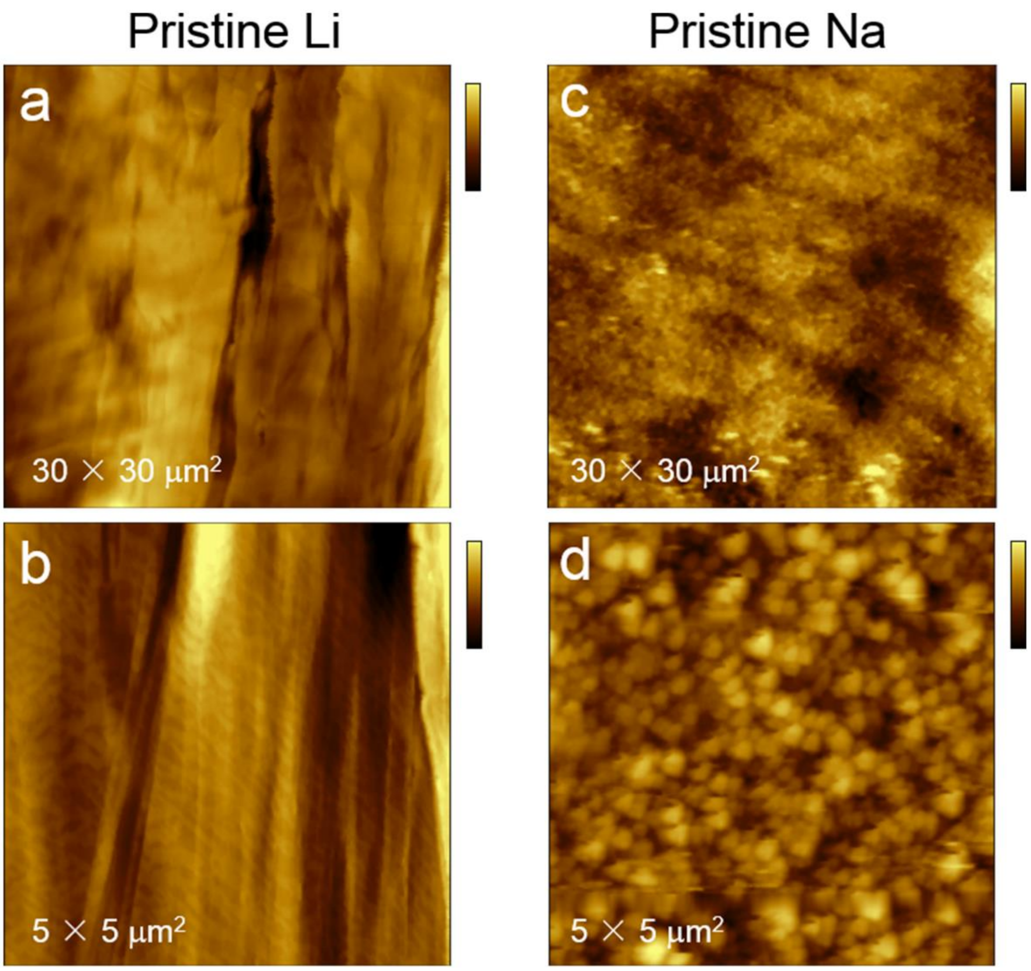

Supplementary Figure 2 | Surface morphologies of pristine Li and Na surfaces. AFM images of pristine Li **(a,b)** and Na **(c,d)** surfaces. Color bars are 0–1.1 μm **(a)** and 0–0.6 μm **(b)**, 0–0.5 μm **(c)**, and 0–0.2 μm **(d)**.

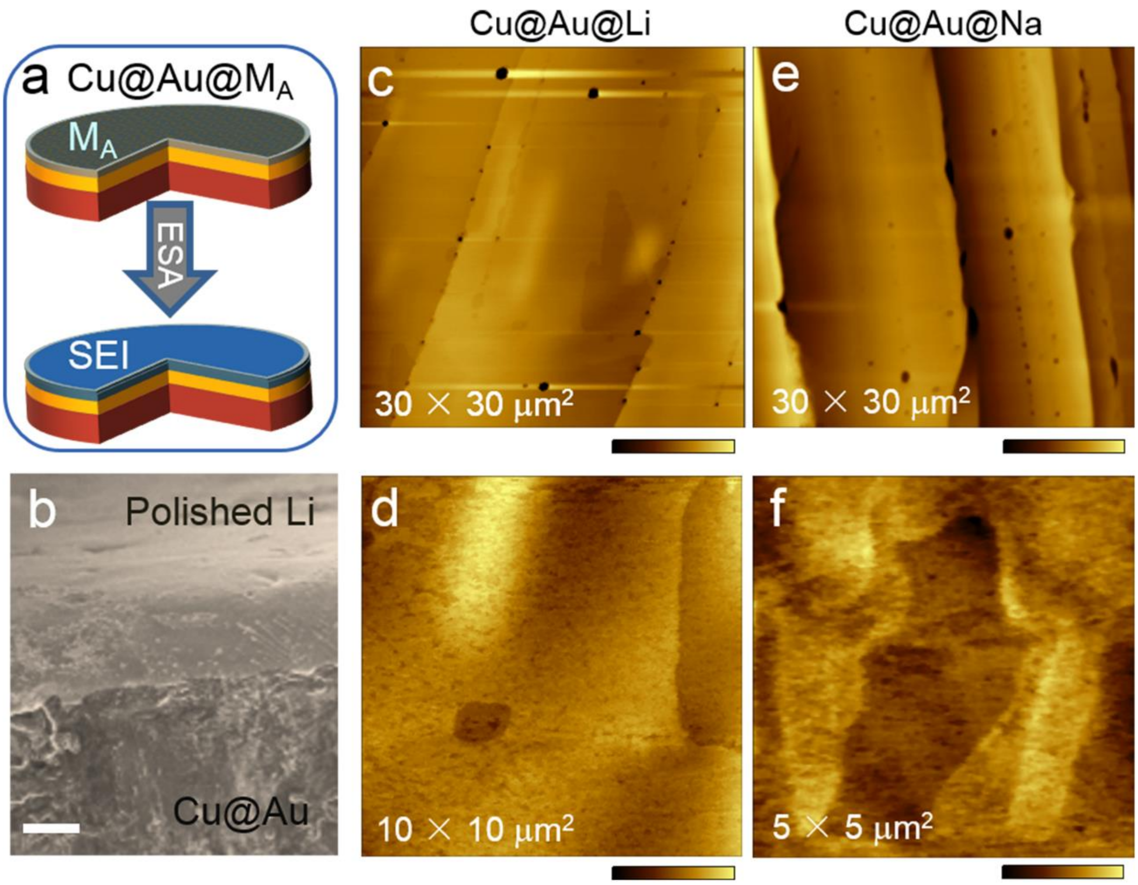

Supplementary Figure 3 | Surface morphologies of electrochemically polished Li and Na thin film. a, Schematic illustration of the Li and Na thin film electrodes. **b,** Cross-section SEM image of the polished Li thin film electrodes. Scale bar: 20μm. After polishing, the thickness of Li thin film is ~25 μm. **c,d,** AFM images of the polished Li thin film surface. Color bars are 0–30 nmm **(c)** and 0–5 nm **(d)**, respectively. **e,f,** AFM images of the polished Na thin film surface. Color bars are 0–0.5 μm **(e)** and 0–4 nm **(f)**, respectively.

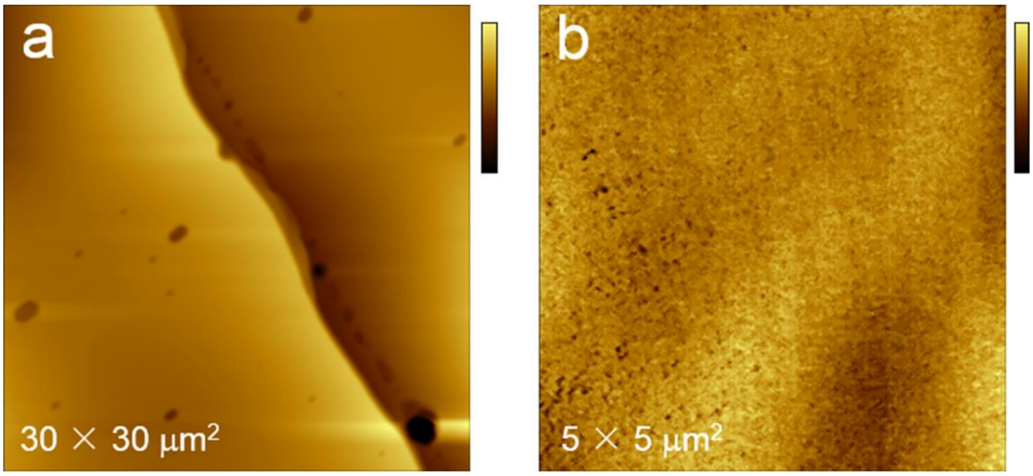

Supplementary Figure 4 | Multi-step electrochemical stripping-annealing strategy for polishing Li surface. a,b, AFM images of Li surface with I-O-I type SEI achieved by a multiple potential step in stripping stage. Color bars are 0–1 μm **(a)** and 0–1.5 nm **(b)**, respectively.

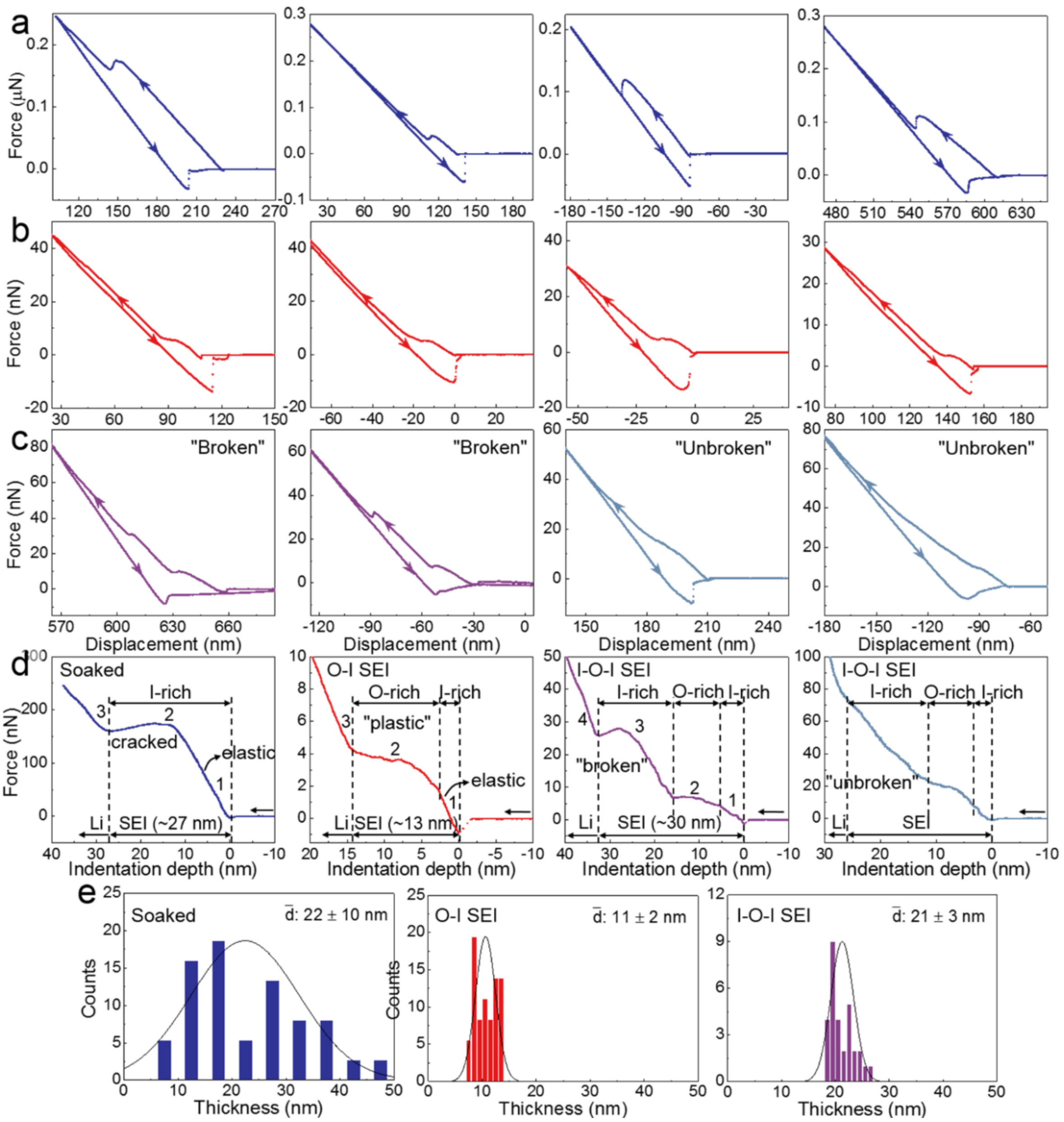

Supplementary Figure 5 | AFM characterization of mechanical properties of the SEI layers. a–c, Typical force-displacement curves of soaked Li surfaces **(a)** and polished Li surfaces with O-I type **(b)** and I-O-I type SEI **(c)**. **d,** Typical force-indentation curves of soaked and polished Li surfaces. **e,** Histogram of thickness of SEI layers constructed from on soaked and polished Li surfaces. For the polished Li electrode with I-O-I type SEI, there are two kinds of force-displacement curves, corresponding to an additional elastic-plastic deformation-like process and an occurrence of a dip.

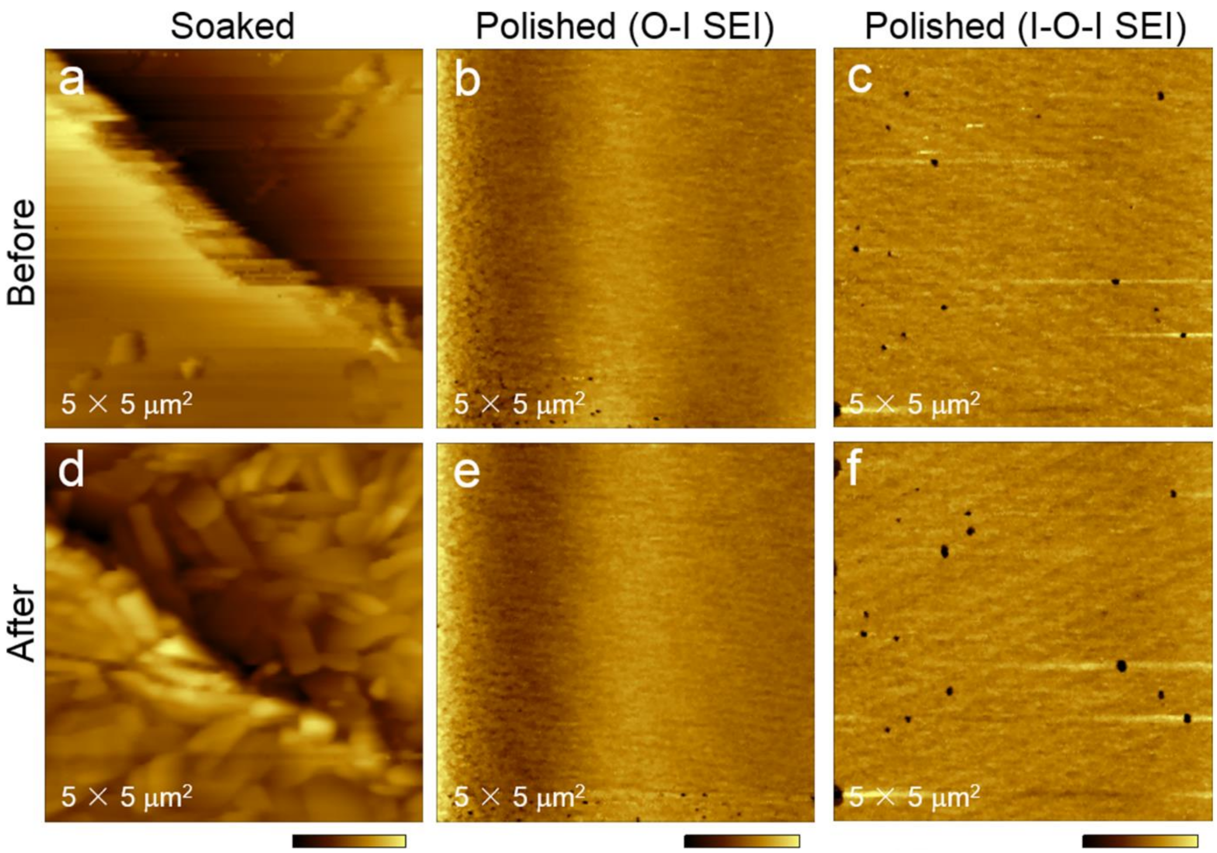

Supplementary Figure 6 | AFM images of polished and soaked surfaces before and after an array of 8 by 8 force curve measurements. The polished surface maintains intact, while the soaked surface breaks into fragments. Color bars are 0–0.5 μm **(a,d)**, 0–1 nm **(b,e)**, and 0–2 nm **(c,f)**.

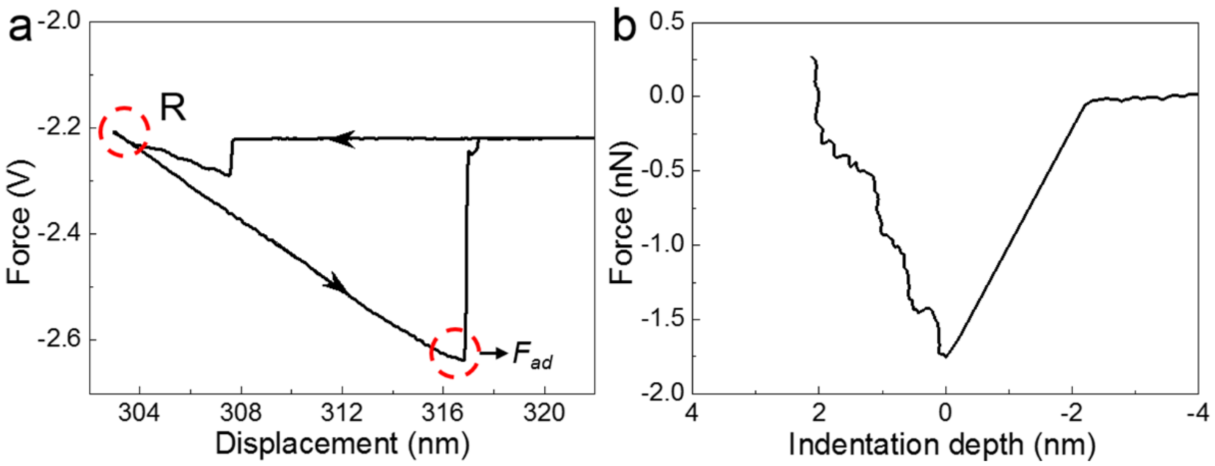

Supplementary Figure 7 | Analysis of adhesion force for polished Li surfaces. a, Force-displacement curve of polished Li surfaces recorded with withdrawing carefully controlled at point "R" in the elastic deformation region. **b,** Force-indentation curve converted from **(a)**. The adhesion force, F_{ad} is also marked with red cycle in **(a)**.

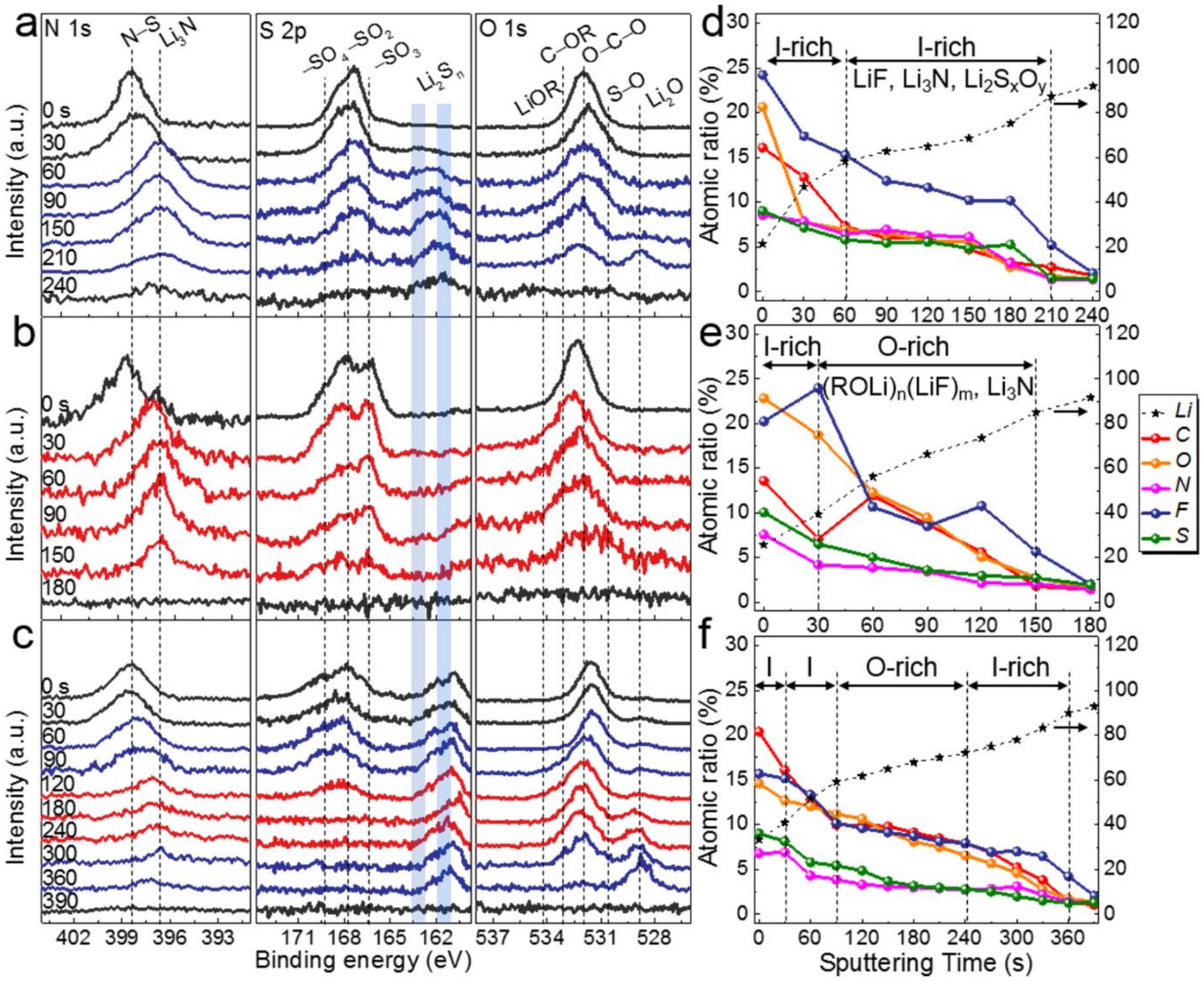

Supplementary Figure 8 | Characterization of chemical composition and structure of SEI layers. a–c, XPS spectra of N 1s, S 2p and O 1s of soaked **(a)** Li surface and polished Li surface with O-I type **(b)** and I-O-I type **(c)** SEI, respectively. **d–f,** The corresponding depth profiles of the atomic concentration of Li, C, O, N, F and S. Focus on the S 2p spectra, the main feature for the polished Li surface with I-O-I structured SEI is the signal of Li_2S (160.9 eV) throughout the thickness, which is a clear difference from the other two samples and suggests the quite different formation mechanism of these electrodes.

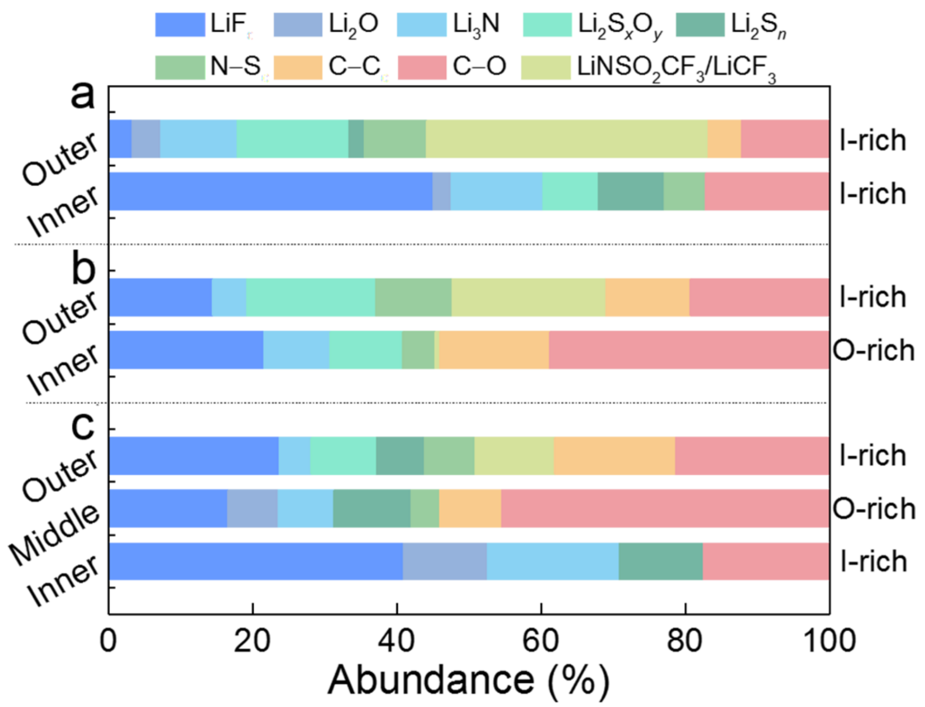

Supplementary Figure 9 | SEI composition of different Li samples. Calculated percentages of some specified compositions of SEI layers based on the XPS spectra at soaked Li surface **(a)** and polished Li surfaces with O-I SEI **(b)** and I-O-I SEI **(c)**, respectively. For the soaked SEI, the percentage of compositions of the outer layer and the inner layer are the summations of those calculated according to the spectra recorded after 0 and 30 s and after 60~240 s sputtering, respectively. For the O-I SEI, the percentage of composition of the outer layer and the inner layer are the summations of those calculated according to the spectra recorded after 0 s and after 30~180 s sputtering, respectively. For the I-O-I SEI, the percentage of composition of the outer layer, the middle layer and the inner layer are the summations of those calculated according to the spectra recorded after 0~90 s, after 120~240 s, and after 300~390 s sputtering, respectively.

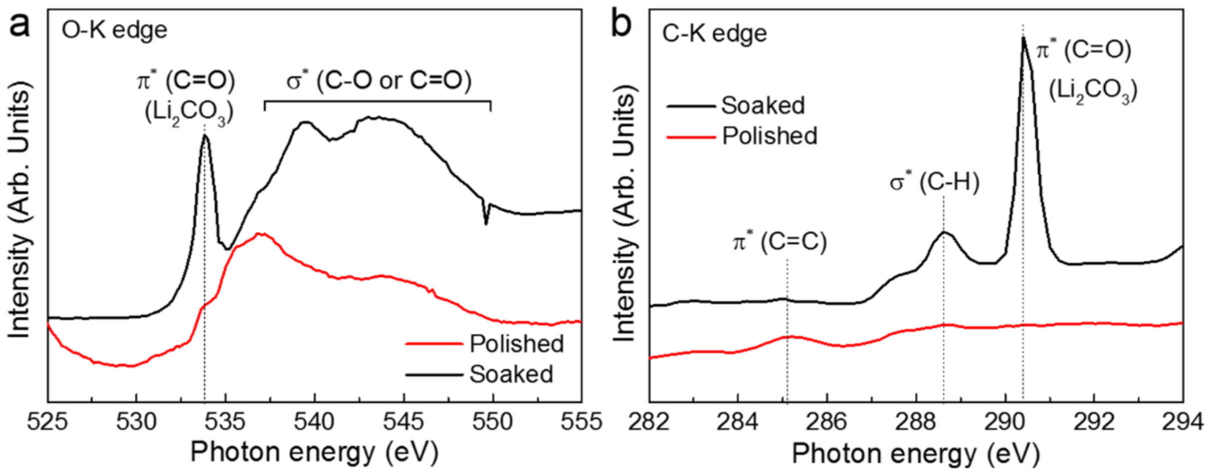

Supplementary Figure 10 | Characterization of chemical composition of SEI layers on different electrodes. O-K edge **(a)** and C-K edge **(b)** XANES spectra of the SEI layers of soaked sample and polished sample with O-I type SEI. Li_2CO_3 exist only in the soaked sample. The broad absorption in the range of 537–550 eV on the O-K spectra suggests the existence of organic molecules in the SEI. The absorption features below 290 eV in the C-K spectra generally manifest organic molecules.

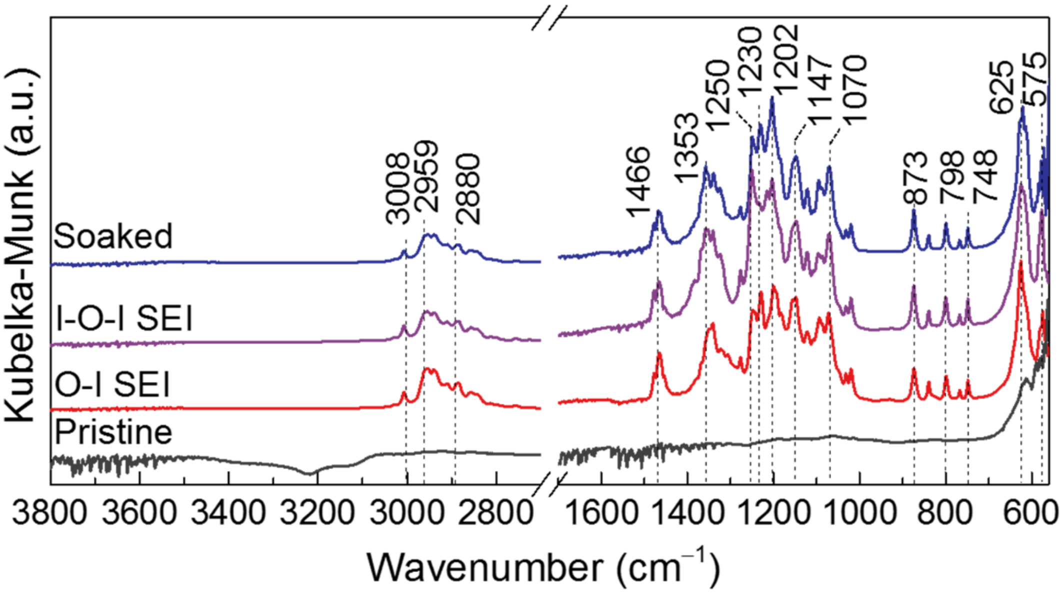

Supplementary Figure 11 | Wide FTIR spectra obtained from pristine, soaked and polished electrode surfaces. For the polished Li electrode with O-I type SEI, the measured FTIR spectrum has two remarkable features of sharp and well-separate bands in the region of 700–900 cm^{-1} and broad background in the region of 1000–1400 cm^{-1}. The band at 625 cm^{-1} is assigned to the Li–OR stretching as well as to Li–F from small $(LiF)_n$ clusters according to reference (Ref. 49 cited in the main text). While for the polished Li electrode with I-O-I type SEI, ν_sS=O band at 1230 cm^{-1} and ρLi–O band at 575 cm^{-1} are the main features distinguish from others. The absence of ν_sS=O band at 1230 cm^{-1} in the I-O-I SEI likely implies that some of the LiTFSI is decomposed to form e.g. Li_2S, in echo with the observation from XPS. The bands at 873, 838 and 798 cm^{-1} of all the samples can be assigned to arise from $Li_2NSO_2CF_3$ and/or $Li_2S_2O_4$ since only these two molecules exhibit a series of band corresponding to S–O stretching mode vibrations in these vibrational frequencies according to the references (Ref. 37 cited in the main text). Detailed band assignments are provided in Supplementary Table 4.

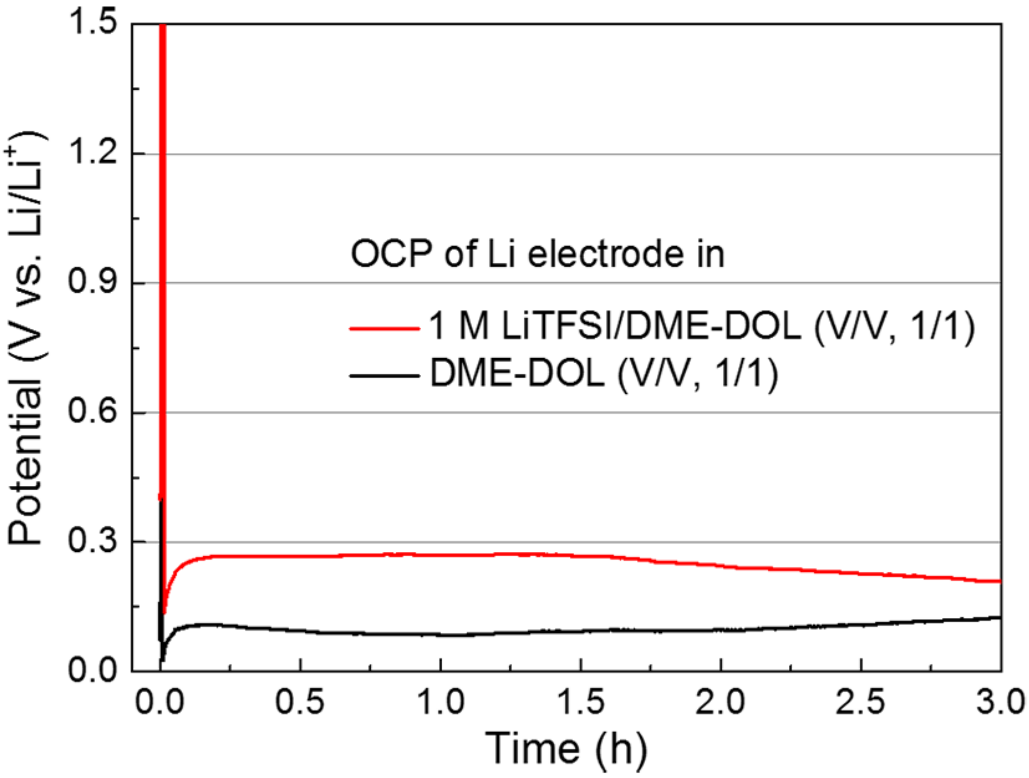

Supplementary Figure 12 | Open circuit potential (OCP) of Li electrode. Li foil was used as the working electrode.

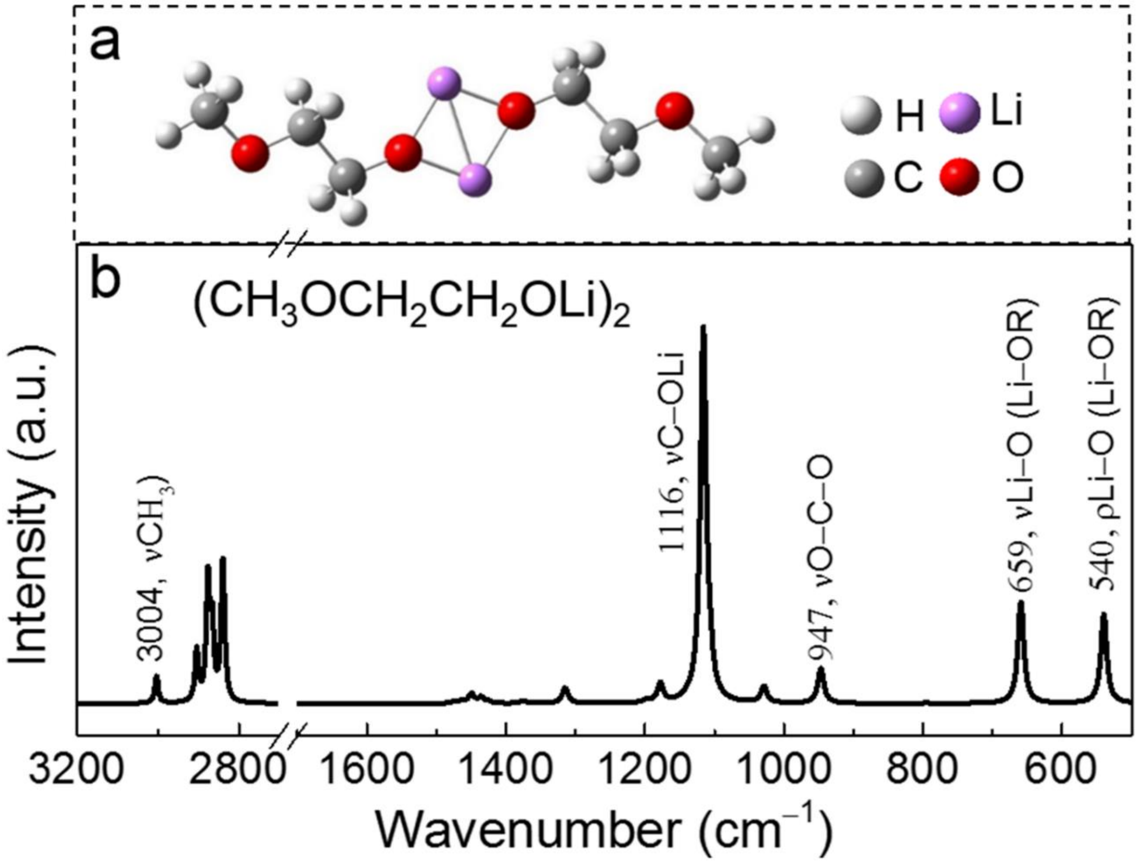

Supplementary Figure 13 | The calculated FTIR spectrum of $(CH_3OCH_2CH_2OLi)_2$ dimer.

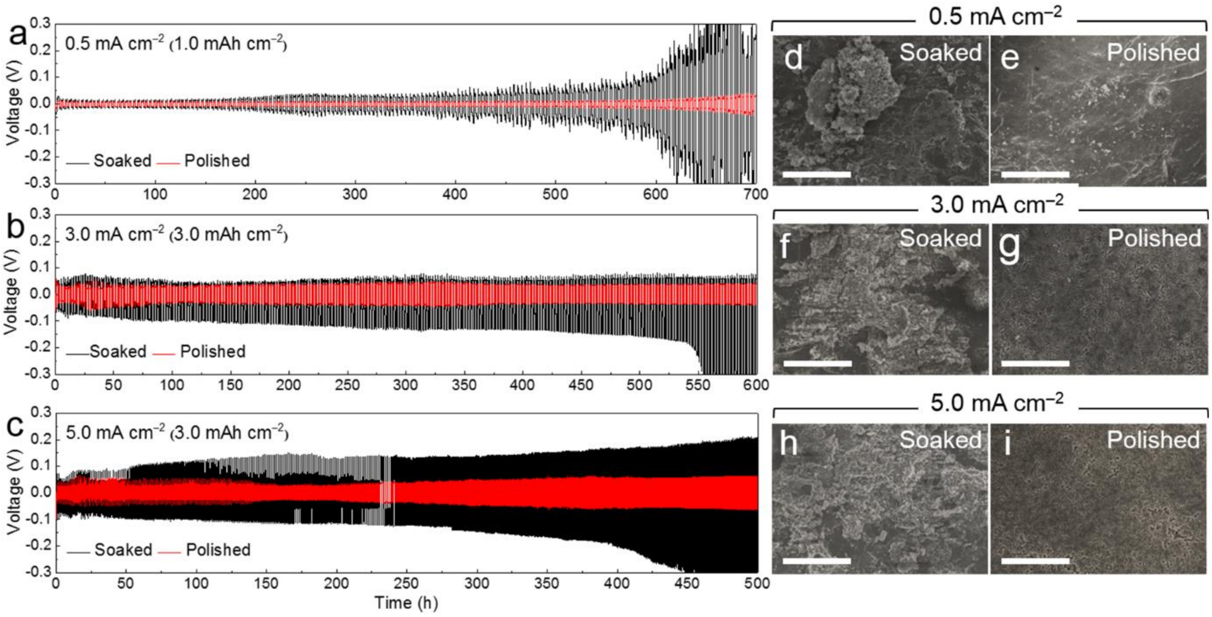

Supplementary Figure 14 | Correlation of cycling behavior and current density. a–c, Voltage profiles for symmetric Li/Li cells at current density of 0.5 mA cm^{-2} (1.0 mAh cm^{-2}) **(a)**, 3.0 mA cm^{-2} (3.0 mAh cm^{-2}) **(b)**, and 5.0 mA cm^{-2} (3.0 mAh cm^{-2}) **(c)**. **d–i,** SEM images of different Li electrodes after 100 cycles. Scale bars in **(d–i)**: 50 μm.

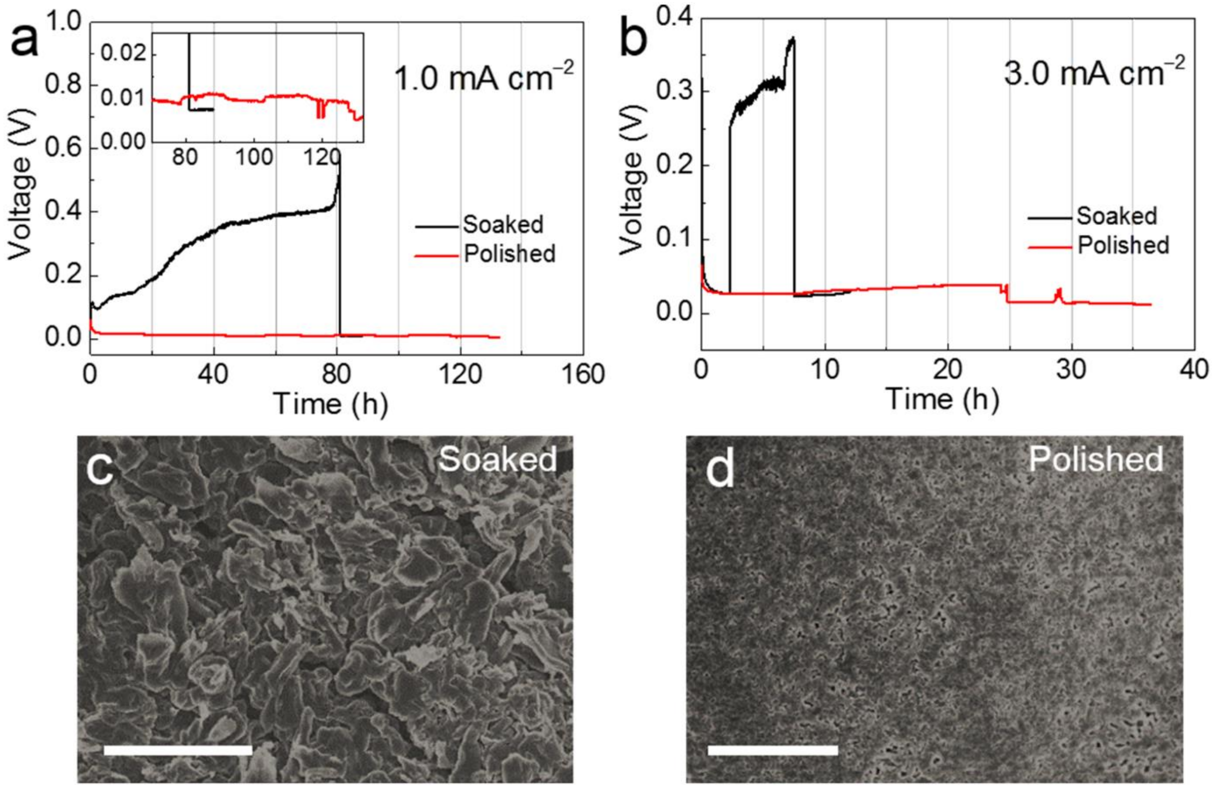

Supplementary Figure 15 | Prolonged polarization behavior of polished Li electrodes. a, b, Voltage profiles of galvanostatic polarization of symmetric Li/Li cells at 1.0 mA cm^{-2} **(a)** and 3.0 mA cm^{-2} **(b)**. **c,d,** SEM images for soaked and polished electrodes after 8 h of polarization at 3.0 mA cm^{-2}. Scale bars on SEM images: 100 μm.

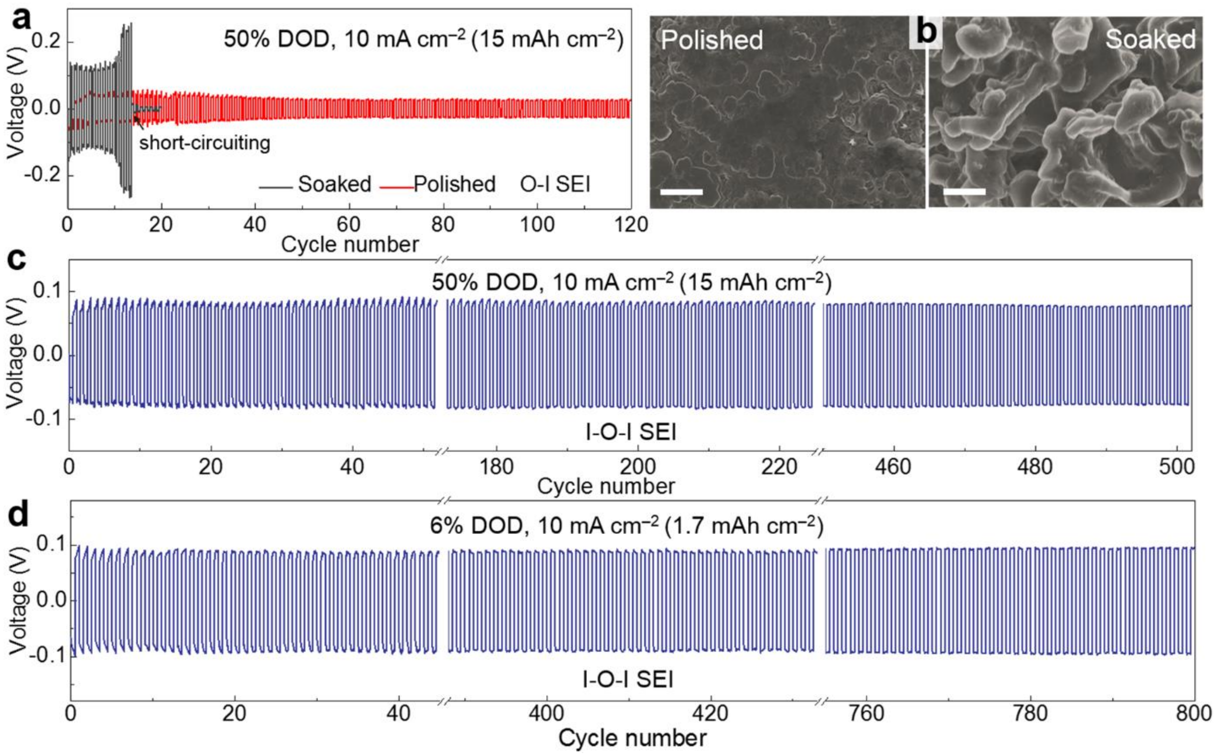

Supplementary Figure 16 | Enhanced electrochemical performance of polished Li anodes. **a,b,** Voltage profiles **(a)** and SEM images **(b)** from symmetric Li cells with soaked Li thin film electrode and polished Li thin film electrode (O-I type SEI) along cycling at 50% DOD in LiTFSI containing electrolyte. Scale bars, 10 μm. **c,d,** Voltage profiles from symmetric Li cells with polished Li thin film (I-O-I type SEI) along cycling at 50% **(c)** and 6% **(d)** DOD.

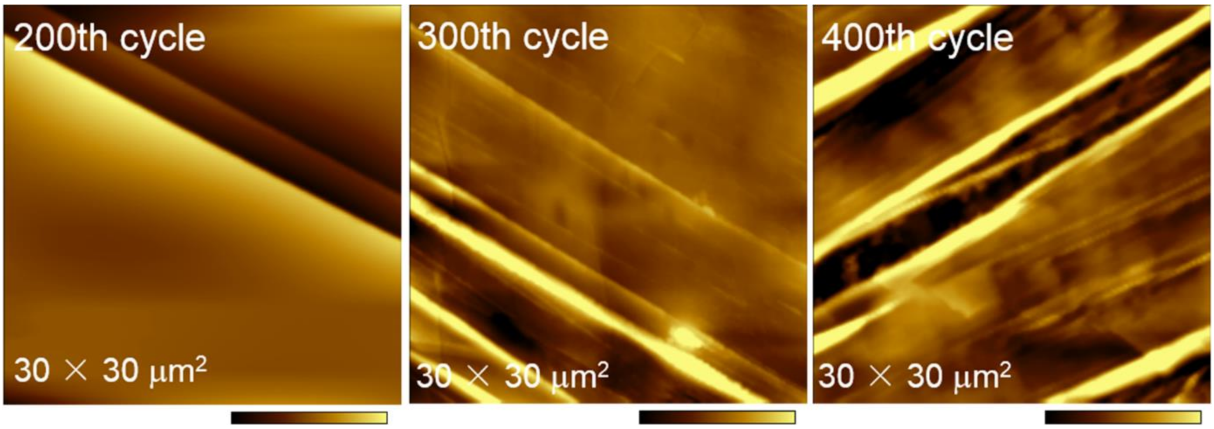

Supplementary Figure 17 | AFM images showing the morphological changes of Li anode with I-O-I type SEI. Electrodes were harvested in the platted state from symmetric Li cells cycled at 10 mA cm^{-2} (3 mAh cm^{-2}) with 1 M LiTFSI in DME-DOL (V/V, 1/1) electrolyte after 200–400 cycles. Color bars, 0–0.3 μm.

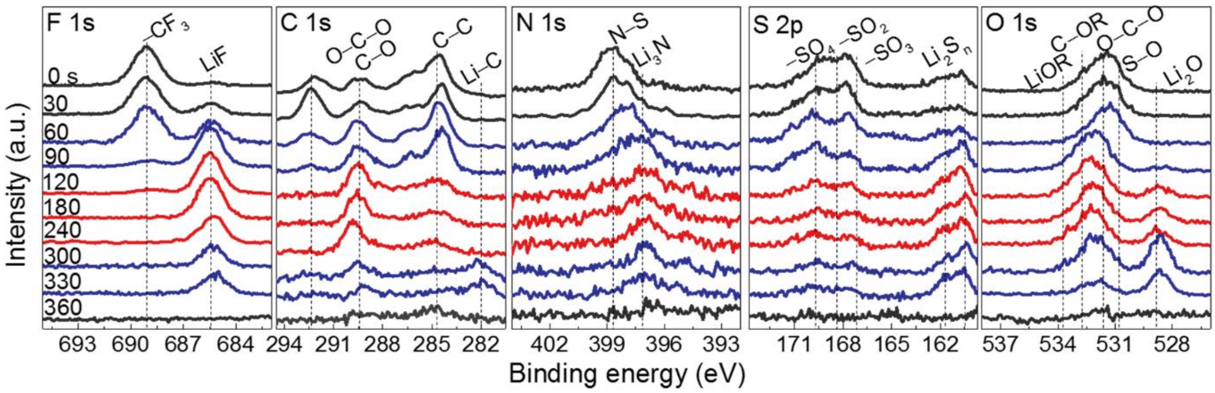

Supplementary Figure 18 | XPS spectra of Li anode with I-O-I structured SEI after 200 cycles. The vertical structure, chemical composition and thickness of the SEI are almost unchanged after 200 cycles, which certainly prove the chemical and structure stability of I-O-I structured SEI.

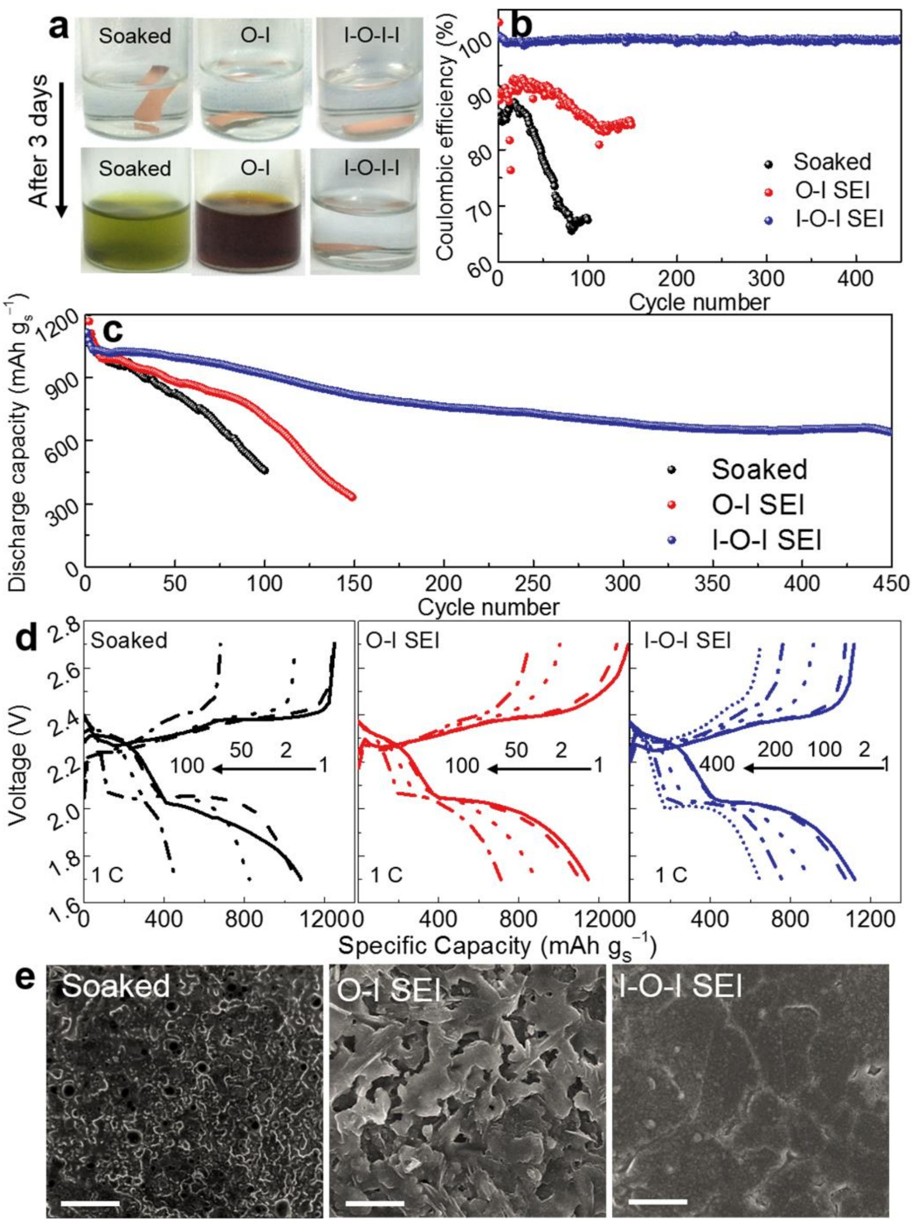

Supplementary Figure 19 | Electrochemical performance of Li-S batteries with different Li thin film electrodes. a, Optical images of untreated, soaked and polished Li thin film soaked in saturated sulfur/DME solution for 2 days. **b–d,** Coulombic efficiency **(b)**, cycling performance **(c)** and voltage profiles of Li-S cells **(d)** of different Li electrodes at 1C. **e,** SEM images of different Li electrodes after 50, 100 and 300 cycles, respectively. Scale bars, 10 μm.

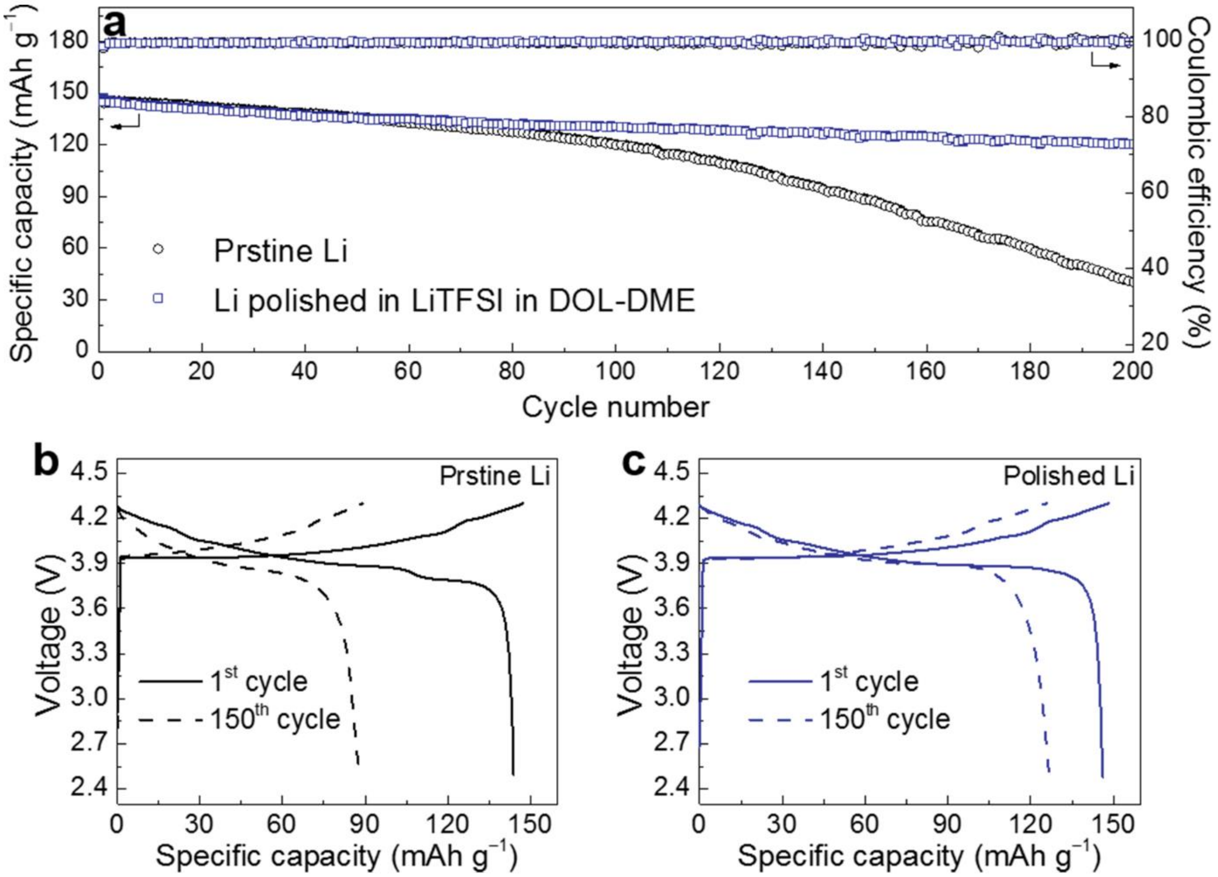

Supplementary Figure 20 | Electrochemical performance of Li||$LiCoO_2$ full cells with different Li anodes . a–c, Cycling performance **(a)** and voltage profiles of the full cells **(b,c)** with different Li anodes at 0.5 C.

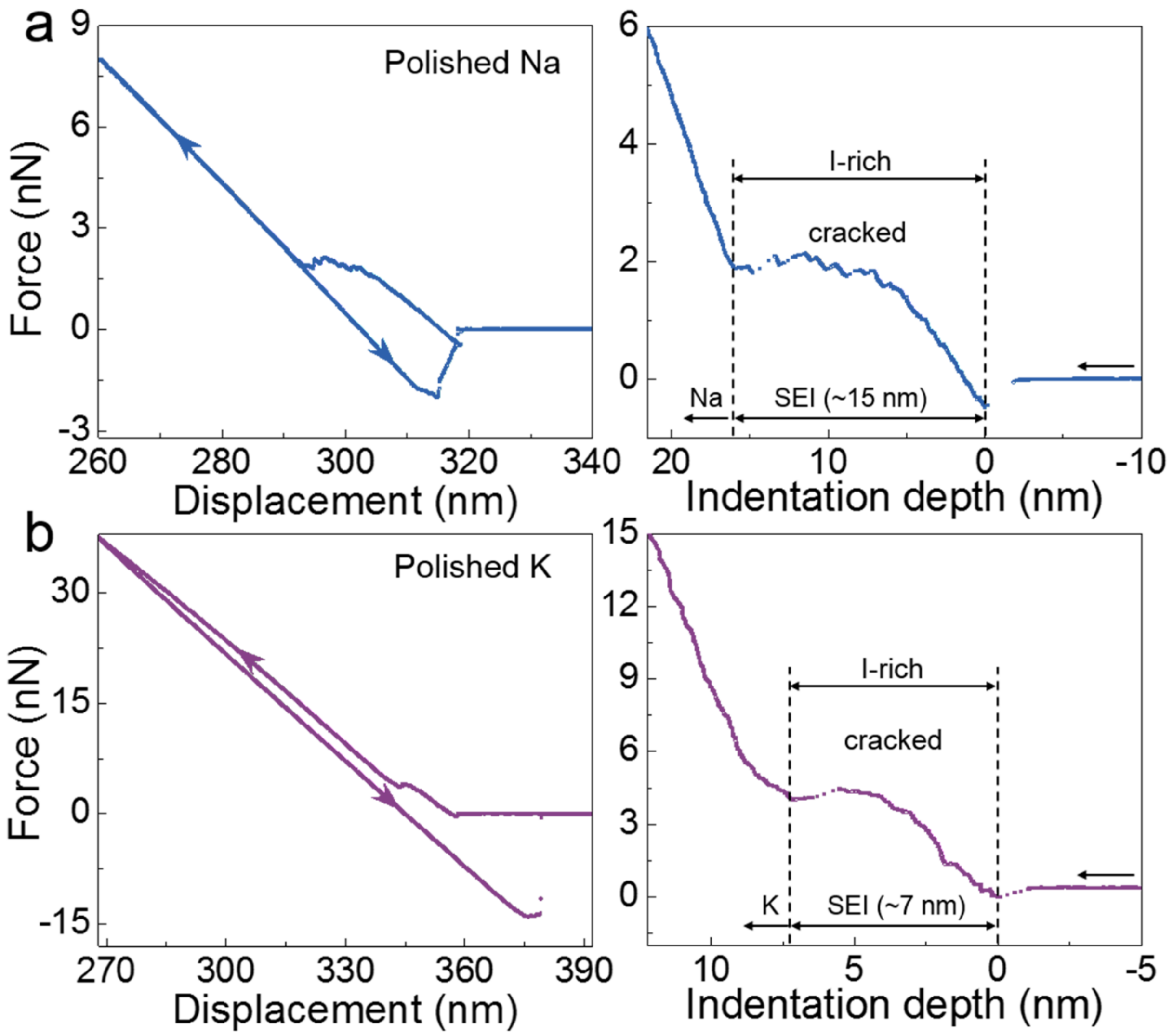

Supplementary Figure 21 | AFM characterization of mechanical properties of the SEI layers on polished Na and K electrodes. a, Typical force-displacement and force-indentation curves of polished Na surface. **b,** Typical force-displacement and force-indentation curves of polished K surface.

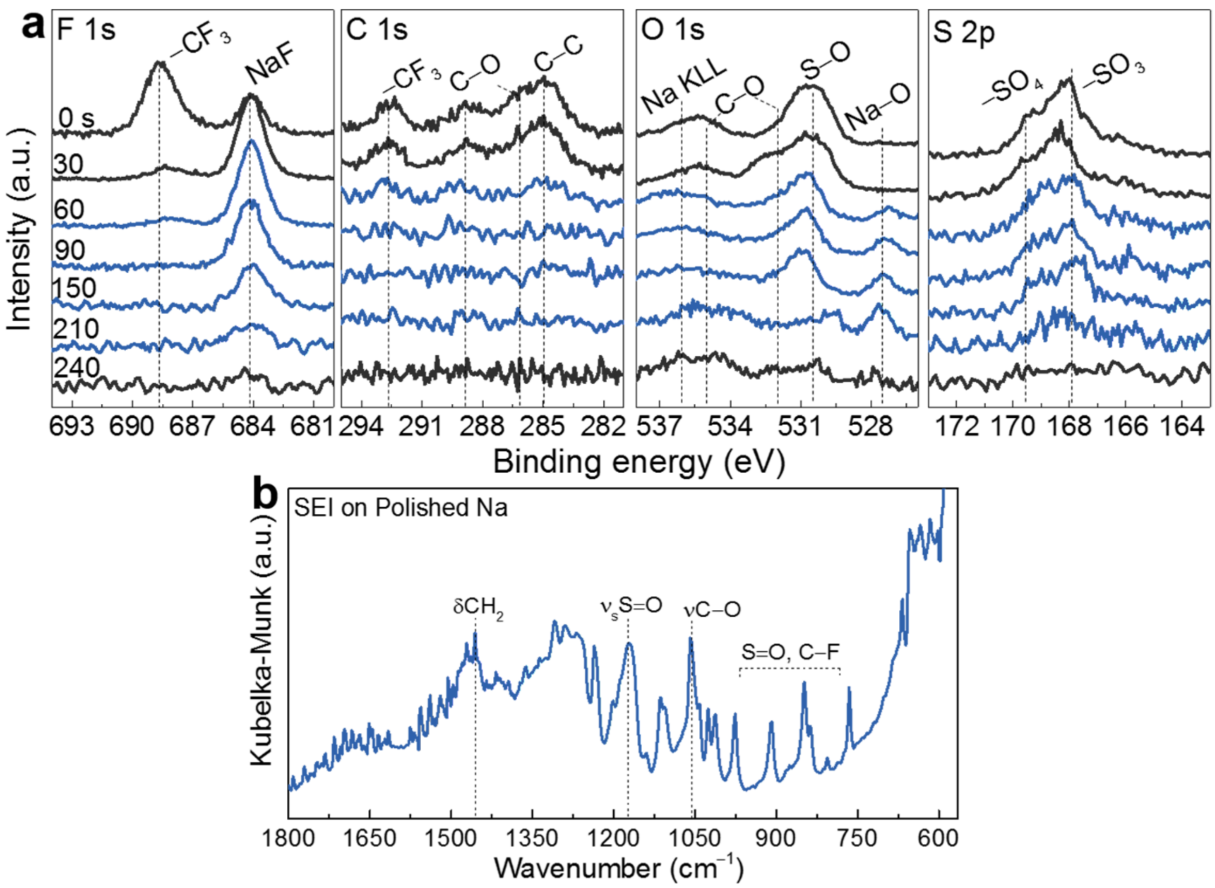

Supplementary Figure 22 | Characterization of chemical composition and structure of SEI layer on polished Na anode. a–c, XPS spectra of F 1s, C 1s, O 1s and S 2p of polished Na surface. **b,** FTIR spectrum for polished Na anode. The inorganic species of NaF (F 1s), Na_2SO_4 (S 2p) are the major components throughout the SEI and Na_2O (O 1s) is present in the inner region, while only very little organic moieties of aliphatic C and C–O (C 1s and O 1s) exists in the surface region. The existences of SO_4^{2-} and C–O fragments are also observed on FTIR spectrum. The clear cut of XPS signals on the spectra between etching time of 30 and 60 s divides the SEI film into two inorganic-rich layers. Both of the XPS and FTIR results confirm that the USUT SEI on polished Na metal (~16 nm) is all-inorganic type.

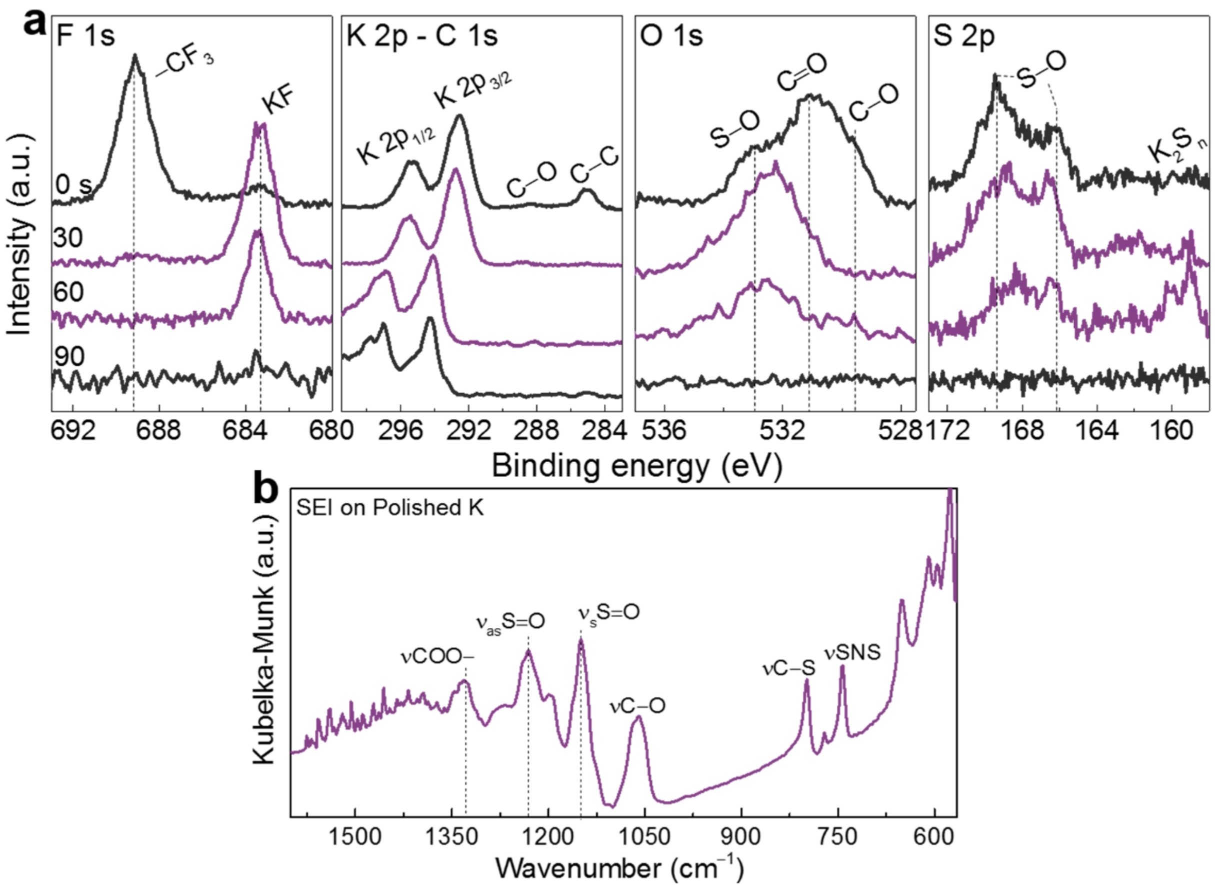

Supplementary Figure 23 | Characterization of chemical composition and structure of SEI layer on polished K anode. a–c, XPS spectra of F 1s, K 2p, C 1s, O 1s and S 2p of polished K surface. **b,** FTIR spectrum for polished K anode. For polished K SEI, the major components are inorganic species including KF, K_2SO_4, K_2S, while organic moieties of aliphatic C and C–O are very little. This confirms that the SEI on polished K anode (~6 nm) is also all-inorganic structured.

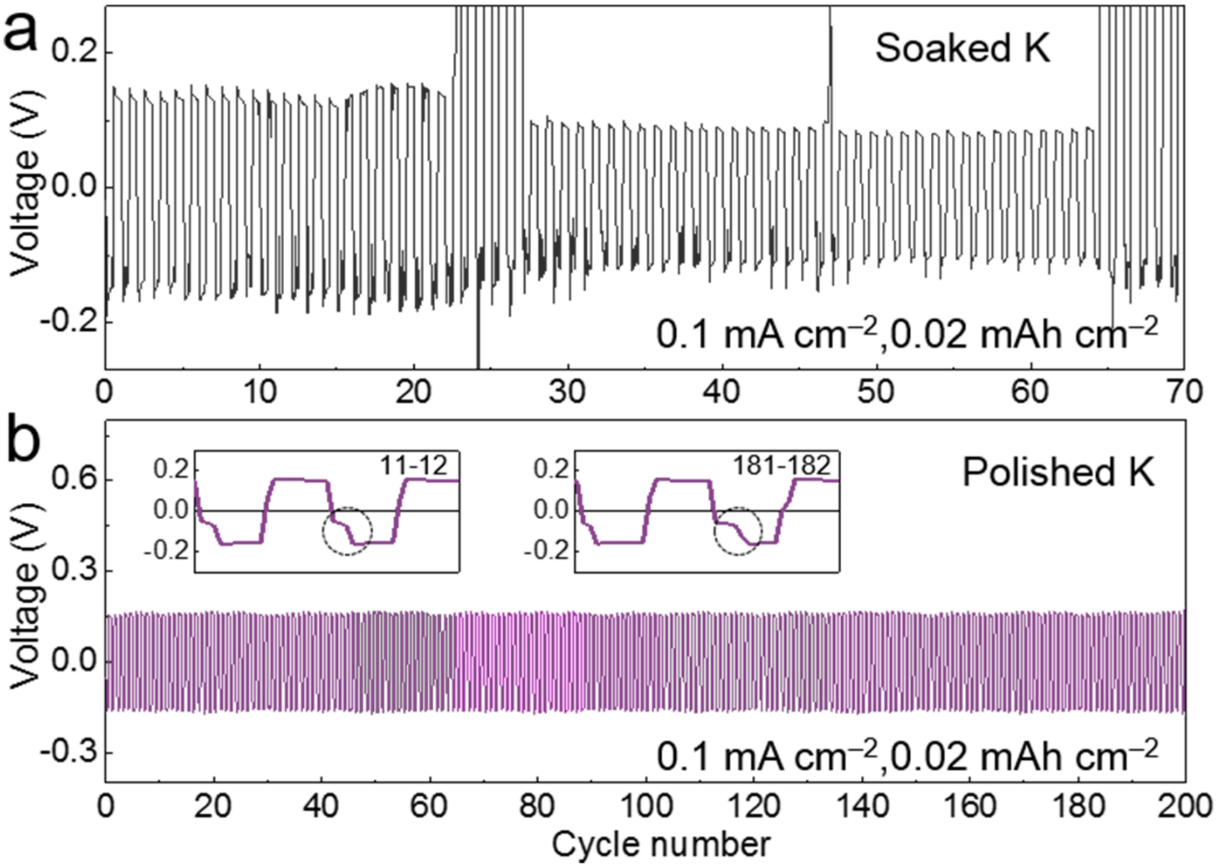

Supplementary Figure 24 | Electrochemical performance of polished K anodes. Voltage profiles of symmetric K cells with soaked **(a)** and polished **(b)** K foil electrodes.

Supplementary Table 1 | Comparison of recent literature reports on cycling performances of Li anodes treated by different strategies.

Strategies		References	Cell performances				SEI structure and properties			
			Cell	Current density & Capacity	Li DOD	Performances	Smoothness / Compactness	Structure / Thickness	Mechanic	Resistance (conductivity)
Artificial	Layered Li–rGO anodes	*Nat. Nanotech.* **11**, 626 (2016)	Li‖Li	3 mA cm^{-2}, 1 mAh cm^{-2}	<1%	100 cycles	Relatively smooth and compact	•N/A •N/A	N/A	~25 Ω
Artificial	Hollow carbon spheres coating	*Nat. Nanotech.* **9**, 618 (2014)	Li‖Li	1 mA cm^{-2}, 1 mAh cm^{-2}	<1%	150 cycles	Compact	•Carbon layer •~500 nm	N/A	~105 Ω (SEI + carbon)
Artificial	3D Cu current collectors	*Nat. Commun.* **6**, 8058 (2015)	Li‖Li	0.2 mA cm^{-2}, 0.5 mAh cm^{-2}	25%	120 cycles	N/A	•Mosaic SEI •N/A	N/A	~30 Ω
Artificial	ALD coating with Al_2O_3	*ACS Nano* **9**, 5884 (2015)	Li‖S	0.14 mA cm^{-2}, 1.4 mAh cm^{-2}	<1%	100 cycles	Compact	•Al_2O_3 layer •14 nm	N/A	~400 Ω
Natural	Dual-salts electrolyte	*J. Power Sources* **271**, 291 (2014)	Li‖Li	10 mA cm^{-2}, 15 mAh cm^{-2}	~10%	180 cycle	N/A	•LiF-rich rigid SEI •N/A	N/A	N/A
Natural	Self-healing (electrostatic shielding)	*J. Am. Chem. Soc* **135**, 4450 (2013)	Li‖ LTO	0.2C	<1%	300 cycles	N/A	•N/A •N/A	N/A	N/A
Natural	Electroplating pretreatment	*Chem* **2**, 258 (2017)	Li‖S	2 mA cm^{-2}, 2 mAh cm^{-2}	<1%	600 cycles	Relatively smooth and compact	•Mosaic SEI •14 nm	N/A	N/A
Natural	Concentrated electrolyte	*Nat. Commun.* **4**, 1481 (2013)	Li‖S	0.2C	<1%	100 cycles	N/A	•Mosaic SEI •N/A	N/A	N/A
Natural	Electrochemical polishing	This work	Li‖Li	10 mA cm^{-2}, 15 mAh cm^{-2}	50%	300 cycles	Ultra-smooth and compact	•Alternating O-rich and I-rich multi-layered structure; •12–27 nm	Coupled rigidity and elasticity	21–28 Ω (3× 10^{-8}–5 × 10^{-8} S cm^{-1})
			Li‖S	2 mA cm^{-2}, 2 mAh cm^{-2}	30%	200 cycles				

*The DOD for Li in these reported literatures are no more than 1% unless otherwise specified, which are estimated according to the 1 cm^2 Li foil with 0.75 mm in thickness. While the DOD in this work for Li|Li and Li|S cells are 50% and 30%, respectively, as described in Methods.

Supplementary Table 2 | Comparison of recent literature reports on cycling performances of Na anodes treated by different strategies.

**Strategies**		**References**	**Cell performances**			
			Cell	Current density & Capacity	Na DOD	Performances
Artificial	ALD coating with Al_2O_3	*Adv. Mater.* 29, 1606663 (2017)	Na||Na	3 mA cm^{-2}, 1 mAh cm^{-2}	<1%	500 cycles
	ALD coating with Al_2O_3	*Adv. Energy Mater.* 7, 1601526 (2017)	Na||Na	0.5 mA cm^{-2}, 1 mAh cm^{-2}	<1%	~30 cycles
	Modified current collectors	*Nano Lett.* 17, 1296 (2017)	Na||Al-C	0.5 mA cm^{-2}, 0.25 mAh cm^{-2}	100%	150 cycles
Natural	New liquid electrolyte	*ACS Cent. Sci.* **1**, 449 (2015)	Na||Cu	0.5 mA cm^{-2}, ~0.6 mAh cm^{-2}	100%	300 cycle
	Concentrated electrolyte	*Nano. Energy* **30**, 825 (2016)	Na||Cu	1 mA cm^{-2}, ~0.6 mAh cm^{-2}	100%	300 cycle
	Concentrated electrolyte	*ChemSusChem.* **10**, 401 (2017)	Na||Cu	0.2 mA cm^{-2}, 0.5 mAh cm^{-2}	100%	300 cycles
Natural	Electrochemical polishing	This work	Na||Na	3 mA cm^{-2}, 1 mAh cm^{-2}	1%	800 cycles
			Na||Cu	2 mA cm^{-2}, 1 mAh cm^{-2}	100%	550 cycles

*The DOD for Na in these reported literatures are estimated according to the ~1 cm^2 Na foil with 1 mm in thickness unless otherwise specified.

Supplementary Table 3 | Li ion surface concentration after Li dissolution of different time.

t / s	Q / mC cm^{-2}	n / mol dm^{-2}	δ / µm	c(Li^+) / mol dm^{-3}
1	200	2.07×10^{-3}	20	**1**
5	1,000	1.03×10^{-3}	45	**2**
20	4,000	4.14×10^{-3}	89	**5**
50	10,000	1.03×10^{-3}	141	**7**
120	24,000	2.48×10^{-3}	219	**11**

Supplementary Table 4 | Vibrational assignments of the main bands of FTIR spectra.

$\nu_{exp.}$ / cm^{-1}	$\nu_{calc.}$ / cm^{-1}	$\nu_{ref.}$ / cm^{-1}	Assignment	References*
3008	3004	—	νCH_3 , ($^*CH_3OCH_2CH_2OLi)_2$	—
2959	2904	—	$\nu_{as}CH_3$ ($^*CH_3OCH_2CH_2OLi)_2$ or other ROLi	—
2880	2877	—	$\nu_s CH_2$, $(CH_3O^*CH_2CH_2OLi)_2$ other ROLi)	—
1466	1450	—	δCH_2, $(CH_3O^*CH_2CH_2OLi)_2$	—
1353	1422	—	ωCH_2, $(CH_3O^*CH_2CH_2OLi)_2$	—
1339	—	1330	$\nu(N–SO_2)$ $(Li_2NSO_2CF_3)$	37
1250	—	1245	$\nu_{as}SO_2$	37
1230	—	1231	$\nu_s SO_2$	37
1202	—	1190	νC–F $(Li_2NSO_2CF_3)$	37
1147	—	—	$\nu O–C(CH_3OCH_2{}^*CH_2{}^*OLi)_2$	—
1095	1116	—	νCOLi	—
1019	1028	—	ν(O–C–C) ($^*CH_3OCH_2CH_2OLi)_2$	—
873, 838, 798	—	700–900	νS–O	37
748	—	755	S–N, N–S–O (LiTFSI)	15
625	656	626	νLiO $(CH_3OCH_2CH_2OLi)_2$ or other ROLi (656 cm^{-1}); $(LiF)_2$ (626 cm^{-1})	46
575	540	—	ρLiO	—

*These references are cited in the main text.

ν = stretching, δ = bending, ω = wagging, ρ = rocking

Supplementary Table 5 | EIS fitting results obtained using the electric equivalent circuit of Fig. 4h in the main text.

Samples		Values
Soaked	R_{SEI} / Ω	87.3
	R_{ct} / Ω	49.52
	Q (Y_0, n) / (10^{-6} $S^n\Omega^{-1}$)	5.91, 0.84
Polished (O-I SEI)	R_{SEI} / Ω	21.51
	R_{ct} / Ω	23.19
	Q (Y_0, n) /(10^{-6} $S^n\Omega^{-1}$)	4.12, 0.86
	ρ / (10^8 Ω cm)	0.32
Polished (I-O-I SEI)	R_{SEI} / Ω	28.35
	R_{ct} / Ω	30.47
	Q (Y_0, n) / (10^{-6} $S^n\Omega^{-1}$)	5.78, 0.82
	ρ / (10^8 Ω cm)	0.19

Supplementary Notes

1. Two-electrode cell vs. three electrode cell for electrochemical polishing

The electrochemical polishing procedure was done in a two-electrode cell. During the potentiostatic stripping, the current density of the working electrode (WE) during could reach up to 200 mA cm^{-2} in the initial stage. But since the counter electrode (CE) was made to have a surface area that is three times of that of the WE, the equivalent current density on the CE is only 70 mA cm^{-2}, with corresponding cathodic overpotential of ca. –150 mV (vs, Li/Li^+). For comparison, the electrochemical polishing was also performed using three-electrode cell. The XPS depth profile show that the SEI prepared in two-electrode and three-electrode cells have similar structure and chemical components, except the thickness of the SEI prepared in the three-electrode cell is slightly thicker by ca. 4 nm at the most. Therefore, the design principle for SEI does not seem to be affected significantly by the polarization of the CE in the two-electrode cell.

2. Estimation of alkali metal ion surface concentration

The substantial increase of alkali metal (M_A) ion surface concentration along with dissolution of M_A surface in the stripping step of the electrochemical polishing benefits the improved surface morphology and desirable SEI. Herein, for example, we estimate the average concentration of Li ion in the vicinity of Li electrode surface during the process of Li dissolution at 1.0 V for different time.

First, the diffusion coefficient of Li^+, D_i, is estimated based on the Nernst-Einstein equation:

$$D_i = \frac{\lambda_i RT}{z_i^2 F^2} \tag{1}$$

where λ_i and z_i are the equivalent molar conductivity and valence number of *i* species, respectively, and F, R and T have usual meanings. Here, λ_i can be obtained from the conductivity σ by the relationship of $\lambda = \sigma_i/c_i$, where c_i is the molar concentration of *i* species in the bulk electrolyte. Given the σ of 15 mS cm^{-1} for 1 mol L^{-3} Li^+ in DOL-DMC (Ref. 21 cited in the main text), the λ of Li^+ is 15 cm^2 S mol^{-1}. This leads to a diffusion coefficient of Li^+ of ca. 4×10^{-6} cm^2 s^{-1}. The value could be reduced if the influence of ionic strength of the medium is taken into account.

Second, the areal charge flux Q and equivalent mole number *n* associated with the stripping process at different time *t* is estimated. To calculate the surface concentration of Li^+ at different states after dissolution takes place, the time-dependent Nernstian diffusion length of Li^+ is estimated using equation of $\delta = \sqrt{(Dt)}$. Thus, the average concentration of Li^+, $c(Li^+)$ can be estimated and the results are listed in the Supplementary Table 3. As can be seen that after dissolution of 120 s, an average concentration of as high as 11 mol L^{-3} can be reached.

3. Calculation of Young's Moduli of SEI films

AFM force curve probes the deformation of sample upon force loading. On the original force-piezo displacement curve, altering of a linear slope means tip encounters an adjacent region with different stiffness. For the convenience of quantitative analysis, the force-displacement curves are converted to force-indentation curves, on which distance deviation brought by tip deformation is removed. Detailed procedure for conversion can be found in reference[4]. Briefly, when a tip runs into contact with the sample surface, the tip-sample distance is coupled with cantilever deflection, z, which should be corrected from the piezo displacement, D, yielding tip indentation depth δ = D – z. The cantilever deflection, z, is obtained by converting deflection signal into unit of distance through the deflection sensitivity specific to the probe spring constant and equipment employed. And then Hooke’s law translates the

cantilever deflection into units of force, leading to force-indentation curve.

The Young's modulus of SEI layer can be estimated based on the elastic deformation region on the force-indentation curve. It is necessary to note, however, the SEIs of both polished and soaked sample are chemically multi-layered. In principle, any Young's modulus of an inner layer can be calculated only if the adjacent outer layer is penetrated so that the tip comes to contact with the inner layer. For the polished sample, the M_A-rich inner layer is buried underneath the outer layer, so it is not possible to exactly estimate its Young's modulus. The situation for the soaked sample is similar except that the SEI cracks before plastic deformation. So for both types of the samples, only apparent Young's modulus can be calculated more accurately from the region with linear slope, i.e. the region with apparent elastic deformation of the SEI. Nevertheless, we also attempted to estimate the Young's modulus of the inorganic-rich inner layer of the I-O-I SEI, despite the value may be influenced by the adjacent outer layer.

In addition, for both the soaked and polished samples, adhesive force is significant, so that Hertz model is no longer applicable to calculate their Young's moduli. Instead, DMT model is used:

$$F + 2\pi R W_{ad} = \frac{4}{3}\frac{E}{1-\nu^2}\sqrt{R\delta^3} \tag{2}$$

where F is the loading force, δ the indentation depth in the elastic deformation region, E the Young's modulus, v the poison ratio (0.5), and R the tip radius (~8 nm). In addition, W_{ad} is the adhesion work per unit area, which can be determined by the adhesion force, F_{ad}, at jump-off-contact point on the force-displacement curve, divided by the contact area that is determined according to the maximum contact radius, a, at the point just before tip withdrawing on the force-displacement curve.

To obtain the adhesion force, F_{ad}, associated with the tip-sample interaction in the elastic deformation region located in the very initial stage of tip indentation, force curves were

recorded with withdrawing point carefully controlled in the elastic deformation region, avoiding tip running into the plastic deformation region. This is shown by Supplementary Fig. 7. The adhesion force, F_{ad}, can be read out from the jump-off-contact, the work of adhesion is calculated by the equation: $W'_{ad} = F_{ad}/2\pi R$[5] and the contact radius, a, calculated by the equation: $a^2 = \delta R$. This allows calculate the work of adhesion per unit area, W_{ad}, and thus Young's modulus by Supplementary Equation (3).

4. Calculation of thickness of SEI based on AFM indentation measurement

Basically, the total thickness of SEI by AFM indentation technique is determined according to the indentation distance up to the point after which the force curve exhibits linear response of indentation. But since Li metal can deform upon indentation as shown by the inclined force-indentation curve in Fig. 2h in the main text (black dotted line for pristine Li metal and purple solid line for the polished Li metal with I-O-I in the deep indentation region), correction is necessary to obtain more accurate thickness values. The correction was done by extrapolating the background-like force-indentation curve due to Li deformation into the region of SEI deformation and then subtracting the indentation depth of Li from that of SEI under the same force value. The corrected force-indentation curves are shown below (right), and statistical analysis suggest total thickness of 21 ± 3 nm for the I-O-I SEI and 11 ± 2 nm for the O-I SEI. These values are slightly smaller than the results obtained from XPS depth profile analysis (12 nm for O-I SEI and 26 nm for I-O-I SEI). We mention that the presence of the unpenetrated and compressed O-I complex (outer) layer leads to smaller thickness; the complex O-I structure also hinders the determination of thickness of each layer of the multilayered SEI. These issues could be hopefully resolved by applying quantitative mechanical modeling and simulation in the future. It is necessary to mention that the extrapolation of the force-indentation curve of Li does not entered the initial stage of the force curve of the two polished

types of Li metal. Therefore the apparent Young's moduli for the complex O-I layers in both O-I and I-O-I SEI remain unchanged.

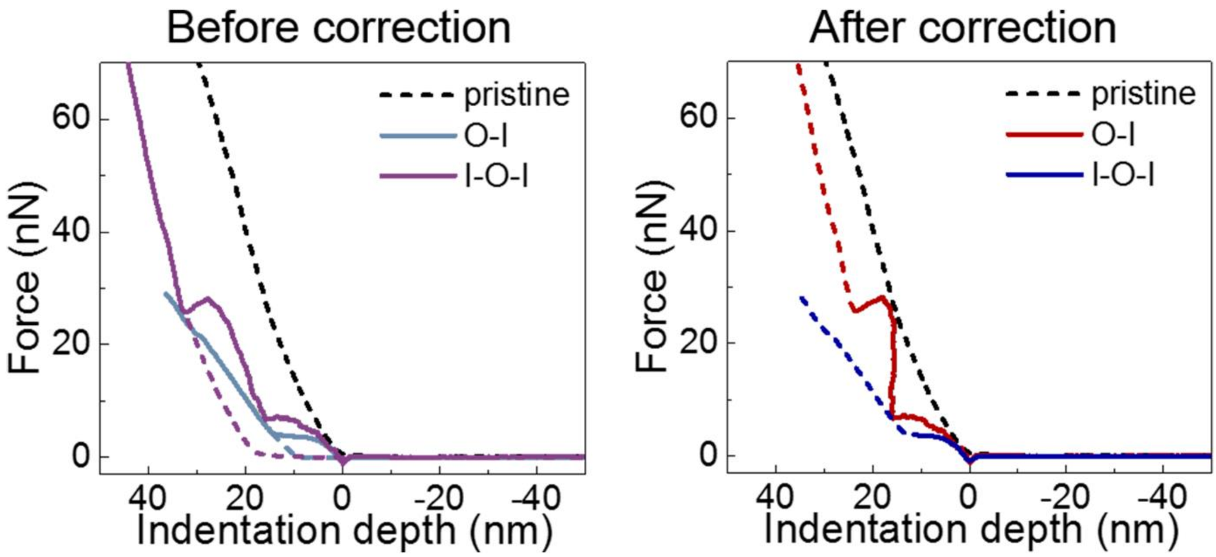

Supplementary Figure | Force-indentation curves of pristine Li and polished Li with O-I and I-O-I structured SEIs before and after the indentation depth introduced by deformation of Li substrate are subtracted.

5. Calculation of the IR spectra and band assignments

IR spectra of a series of free molecules and their dimers were calculated with Gaussian 09 D.01 package[1] using B3LYP functional[2,3]. The basis sets 6-311++G (d, p) were employed for geometry optimization of the molecular structures. No imaginary frequencies were present after optimization. Theoretical vibrational frequencies are scaled by a factor of 0.967. Calculated IR spectra of $CH_3OCH_2CH_2OLi$ dimers are presented in Supplementary Fig. 12. Band from anion reduction products are assigned taking the information from literature works as reference. Detailed band assignments are given in Supplementary Table 4. Although the free molecules, even their dimers, could be very different from those in the SEI layers, the similarities between the calculated and measured spectra allow us to propose that the $CH_3OCH_2CH_2OLi$ may be essential moiety in the SEI layer.

6. Proposed mechanism for electrochemical polishing of M_A surface and formation of multi-layer structured SEIs

The electrochemical polishing of M_A surface, involving anodic dissolution as the first step (stripping step), falls within the framework of general principle for electrochemical polishing of metallic surfaces in aqueous solutions, but distinct with the concurrent reduction of electrolyte and formation of insoluble SEI film which is indispensable for protecting M_A surface after the polishing. A mechanism involving coupled viscous liquid layer[6] and SEI thin film is proposed to account for the polishing of the M_A surface and formation of SEI. In the following, we provide a further discussion on the mechanism of two-step based electrochemical polishing, taking Li metal surface as an example.

In the stripping step, high-rate Li dissolution takes place, which generates an exceedingly high surface concentration of Li^+ so that a viscous layer is formed in the vicinity of the Li surface. The viscous layer is thinner at protrusions than at depressions due to difference in diffusion rate, favoring the preferential dissolution of Li from the protrusions. A primary SEI thin film is formed through electrolyte reduction, with the aid of high Li^+ concentration. DOL can be reduced to $CH_3OCH_2CH_2OLi$ and $CH_3CH_2OCH_2OLi$:

$$C_3H_6O_2 + Li + e^- \rightarrow CH_3OCH_2CH_2OLi + CH_3CH_2OCH_2OLi \quad (ROLi) \qquad (3)$$

where R denotes $CH_3OCH_2CH_2OLi$ and $CH_3CH_2OCH_2OLi$. TFSI anion can be reduced to form various products of different oxidation states:

$$\left(CF_3SO_2\right)_2 N^{-1} + \left(n+1\right) Li^+ + ne^- \rightarrow LiF + LiCF_3 + Li_3N + Li_2SO_2CF_3 + Li_2S_2O_4 + Li_2SO_3 + Li_2SO_4 \quad (4)$$

Depending on the stripping potential (Supplementary Fig. 1), the sequence of reactions and amount of organic and different inorganic products could vary considerably so that chemical composition of the SEI can be manipulated. At an optimum stripping potential, e.g. 1.0 V in the system of DOL/DME-LiTFSI, both the reduction of DOL and TFSI can proceed

moderately to form oligmers of $(ROLi)_n$ which may be cross-linked by small inorganic molecules such as LiF and Li_3N to create a network structure of organic-rich inner layer of SEI. The proposed reactions are as follows:

$$\mathrm{ROLi} \rightarrow (\mathrm{ROLi})_n \tag{5}$$

$$(\mathrm{CF_3SO_2})_2\mathrm{N}^{-1} + (n+1)\mathrm{Li}^+ + ne^- \rightarrow \mathrm{LiF} + \mathrm{LiCF_3} + \mathrm{LiN_3} + \mathrm{Li_2SO_4}\,(\mathrm{or\,Li_2SO_3}) \tag{6}$$

$$(\mathrm{ROLi})_n + (m\mathrm{LiF} + m'\mathrm{Li_3N}) \rightarrow (\mathrm{ROLi})_n \cdot (\mathrm{LiF})_m \cdot (\mathrm{Li_3N})_{m'} \tag{7}$$

A possible structure with network-like organic-rich inner layer and inorganic-rich outer layer, i.e. the O-I type structured SEI, is schematically illustrated in the figure shown below. Naturally, other types of SEI can be easily achieved by multi-step stripping-annealing process.

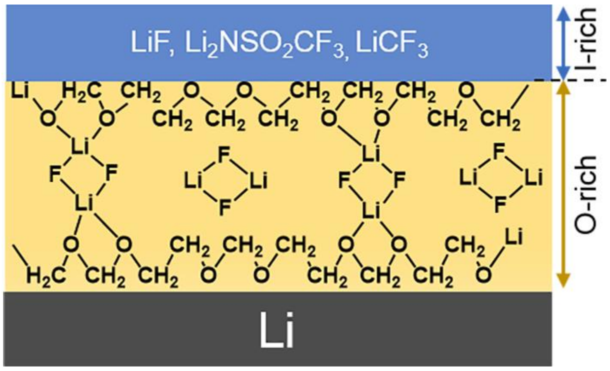

Supplementary Figure | Schematic of a possible SEI structure with network-like organic-rich inner layer and inorganic-rich outer layer, i.e. the O-I type of SEI structure.

7. The analysis of conductivity for M_A^+-rich SEI

The EIS spectra of different electrodes were fitted using the equivalent circuit proposed in Fig. 4h in the main text. In the equivalent circuit, R_s is the bulk resistance of the cell, which includes contributions from the electrolyte, separator and electrodes; R_{SEI} and Q_{SEI} are the resistance and capacitance of the SEI layer, respectively; R_{ct} and Q_{dl} are the Faradic charge-transfer resistance and corresponding double layer capacitance at Li/SEI interface; W is the

Warburg impedance related to a combination of diffusional process of Li^+ across the Li/SEI interface.

Equation $R_{SEI} = \rho l/A$ was used to estimate the resistivity of SEI layer, where l is the thickness of the SEI layer suggested by XPS results, A is the electrode surface area, which is considered unchanged during cycling, and ρ is the resistivity of the SEI. The corresponding conductivity σ of SEI is obtained from ρ.

Supplementary Methods

Reagents and materials. The lithium (Li) metal foils (Φ15.8 × 1.0 mm, Φ22.0 × 0.8 mm, ≥99.9%) used in this work were purchased from Shunyou Metal Co., Ltd. (Shanghai, China). The sodium (Na) and potassium (K) metal foils were prepared by cleaning and flattening the Na metal ingot (Alfa Aesar, ≥99.8%) and K metal cubes (Sigma-Aldrich, ≥99.8%, stored in mineral oil), respectively. Lithium bis(trifluoromethanesulphonyl)imide (LiTFSI, Sigma-Aldrich, ≥99.95%) was used as received without further purification. 1,2-dimethoxyethane (DME, <8 ppm H_2O) and 1,3-dioxolane (DOL, <8 ppm H_2O) were acquired from Smoothway (Shenzhen, China). 1 M sodium trifluoromethanesulfonat ($NaSO_3CF_3$) in diglyme and 1 M KTFSI in DME were obtained from Fosai New Materials Co., Ltd. (Suzhou, China). All regents and materials were handled in Ar-filled glove box (Vigor Tech, O_2 and H_2O < 1 ppm).

Atomic force microscopy (AFM). AFM measurements were conducted on a scanning probe microscope (SPM 5500, Keysight Technologies) inside an Ar-filled glove box. For topographic imaging, contact mode was employed, and a typical scanning rate of 1.5 Hz and loading force of ~2.5 nN were used. For force curve measurements, AFM probes with spring constants of 0.65, 1.41 and 2.75 N m^{-1} were used with typical force loading rates of 625, 800 and 1000 nm·s^{-1} for different type of polished and soaked samples, or otherwise indicated in the main text. Deflection sensitivity of the detector was calibrated using Si wafer.

X-ray photoelectron spectroscopy (XPS) and X-ray absorption spectroscopy. XPS measurements were performed on PHI 5000 Versa Probe II (UlVAC-PHI) using monochromatic Al Kα (1486.6 eV) X-ray source at 24 W and 16 KV with a beam spot size of 100 μm. The binding energies were referenced to the C 1s line at 284.6 eV from adventitious carbon. Depth profiling was fulfilled using Ar ion sputtering in the x-y scan mode at ion acceleration of 2 kV and ion beam current of 2 μA over an area of 2 × 2 mm^2. The thickness of the SEI was estimated based on the calibrated sputtering rate of 5 nm per minute for Si. X-ray absorption near-edge structure spectroscopy (XANES) experiments were performed at the Soft X-ray Spectroscopy

station (BL08U1A) of Shanghai Synchrotron Radiation Facility (SSRF). The electron beam energy of the storage ring was 3.5 GeV with a maximum stored current of 300 mA. The spectra were normalized to the incident photon flux. Thin film type of samples (Cu@Au@SEI) was used, which were obtained by electrochemical polishing of Li thin film coated Cu@Au electrodes after thoroughly removing residual Li by electrochemical dissolution. All Samples were washed thoroughly using the anhydrous DME solvent to remove residual electrolytes, and then transferred into the XPS or XANES chambers by using commercial air-isolating containers.

Fourier transform infrared (FTIR). FTIR measurements were performed on a Nicolet Avatar FTIR Spectrometer (Thermo Scientific) equipped with an MCT detector with a resolution of 4 cm^{-1}. All samples were washed thoroughly using anhydrous DME solvent to remove residual electrolytes and then placed in a sealed and Ar-filled FTIR cell without exposure to air. FTIR spectra were recorded in diffuse reflectance mode. Interferences from CO_2 and H_2O in air are suppressed by filling the testing chamber of the equipment with Ar. Spectra ranging from 400–4000 cm^{-1} with 32 accumulative scans were obtained. All FTIR dates were calibrated by Kubelka-Munk function before analysis.

Other related characterizations. The open circuit potentials (OCP) of Li in DME/DOL (1/1, v/v) solvent and Li in 1 M LiTFSI/DME/DOL (1/1, v/v) electrolyte were measured using Li foil as working electrode. The OCP was read against a Pt wire whose potential was calibrated with respect to a Li foil electrode after prolonged soaking in 1 M LiTFSI/DME/DOL (1/1, v/v) electrolyte. The electrochemical impedance spectra (EIS) were measured before and after charge-discharge cycling using Autolab PGSTAT204 electrochemical analyzer (Metrohm) in the frequency range from1 MHz to 0.01 Hz with peak-to-peak amplitude of 5 mV. The morphologies of metal surfaces before and after cycling were characterized by scanning electron microscope (SEM, SU4800). For morphology measurement after cycling, cells were disassembled in the glove box followed by thoroughly washing the electrodes with anhydrous

DME to remove residual electrolytes. To avoid air exposure of samples, the dried samples were sealed in air-isolating containers in glove box and transferred quickly into the SEM equipment under the protection of Ar flow. Surface element analysis was performed by means of an energy dispersive X-ray fluorescence spectrometer (EDX). Optical images of the samples in this paper were obtained by 8 megapixel Apple iPhone 5s camera from a fixed position under constant lighting conditions.

Supplementary References

1. Frisch M. J., Trucks G. W., Schlegel H. B., Scuseria G. E., Robb M. A., Cheeseman J. R., Scalmani G., Barone V., Mennucci B., Petersson G. A., Nakatsuji H., Caricato M., Li X., Hratchian H. P., Izmaylov A. F., Bloino J., Zheng G., Sonnenberg J. L., Hada M., Ehara M., Toyota K., Fukuda R., Hasegawa J., Ishida M., Nakajima T., Honda Y., Kitao O., Nakai H., Vreven T., Montgomery J. A., Jr., Peralta J. Ogliaro E., Bearpark F., M., Heyd J. J., Brothers E., Kudin K. N., Staroverov V. N., Keith T., Kobayashi R., Normand J., Raghavachari K., Rendell A., Burant J. C., Iyengar S. S., Tomasi J., Cossi M., Rega N., Millam J. M., Klene M., Knox J. E., Cross J. B., Bakken V., Adamo C., Jaramillo J., Gomperts R., Stratmann R. E., Yazyev O., Austin A. J., Cammi R., Pomelli C., Ochterski J. W., Martin R. L., Morokuma K., Zakrzewski V. G., Voth G. A., Salvador P., Dannenberg J. J., Dapprich S., Daniels A. D., Farkas O., Foresman J. B., Ortiz J. V., Cioslowski J., and J. Fox D. Gaussian 09, Revision D.01, Gaussian, Inc., Wallingford CT (2013).
2. Lee, C., Yang, W. & Parr, R. G. Development of the Colle-Salvetti correlation-energy formula into a functional of the electron density. *Phys. Rev. B* **37**, 785–789 (1988).
3. Becke, A. D. Density−functional thermochemistry. III. The role of exact exchange. *J. Chem. Phys.* **98**, 5648–5652 (1993).
4. Senden, T. J. Force microscopy and surface interactions. *Curr. Opin. Colloid Interface Sci.* **6**, 95–101 (2001).
5. Butt, H.-J., Cappella, B. & Kappl, M. Force measurements with the atomic force microscope: Technique, interpretation and applications. *Surf. Sci. Rep.* **59**, 1–152 (2005).
6. Jacquet, P. A. Electrolytic and chemical polishing. *Int. Mater. Rev.* **1**, 157–238 (1956).
